# LumaCam: a novel class of position-sensitive event mode particle detectors using scintillator screens

**DOI:** 10.1038/s41598-024-82095-2

**Published:** 2024-12-16

**Authors:** Alexander Wolfertz, Alex Gustschin, Michael Schulz, Alexander M. Long, Anton Khaplanov, Tsviki Y. Hirsh, Andrei Nomerotski, Manuel Morgano, Anton Tremsin, Giacomo Mauri, G. Jeff Sykora, Adrian Losko

**Affiliations:** 1 Forschungs-Neutronenquelle Heinz Maier-Leibnitz, 85748 Garching, Germany; 2 Los Alamos National Laboratory, Los Alamos, NM 87545 USA; 3 Oak Ridge National Laboratory, Oak Ridge, TN 37830 USA; 4 Soreq Nuclear Research Center, 81800 Yavne, Israel; 5 Czech Technical University, 160 00 Prague, Czech Republic; 6 European Spallation Source ERIC, 22484 Lund, Sweden; 7 Paul Scherrer Institut, 5232 Villigen, Switzerland; 8 Faculty of Materials Engineering, Lund University, 22100 Lund, Sweden; 9 University of California, Berkeley, CA 94720 USA; 10 ISIS Department, Science and Technology Facilities Council, Rutherford Appleton Labs, Didcot, OX11 0QX UK

**Keywords:** Experimental particle physics, Imaging techniques, Characterization and analytical techniques

## Abstract

A new type of position-sensitive detectors is gaining attention in the neutron community. They are scintillator based detectors that detect the scintillation light on an individual photon basis via an image intensifier and a fast image sensor. Their readout operates in event mode i.e. it produces information about individual neutron interactions, reconstructed from the sensor data, thus enabling to achieve superior spatial and temporal resolutions compared to regular detectors. Although the development of current detectors is focused on neutrons, the concept is also applicable to the detection of other particles such as high-energy photons. This document provides a description on how these detectors are built, how they operate, and what their characteristics are. An example of a detector implementation based on a Timepix3 chip is described to illustrate the detector concept. This includes a detailed description of the algorithm that reconstructs the neutron interactions from the sensor data, one of the core components that sets it apart from established scintillator-based imaging detectors. Energy-resolved epithermal neutron radiography was performed at the ISIS EMMA beamline with this detector, illustrating some of the fundamental differences in the data that can be produced with the new type of detector compared to more established types of scintillator based neutron detectors. The term LumaCam is proposed to refer to this new class of position-sensitive event-mode detectors.

## Introduction

In recent years, a new type of position-sensitive neutron detector has started to be developed^1^. Their structure is similar to traditional scintillator based neutron cameras, capturing light from a scintillator screen with an image sensor to obtain a 2D neutron image^2,3^. However, this new type of detector operates in event mode. This means that, the detector output is a list of recognized particle events containing the time and position information of their interaction with the active area of the detector. This type of data output is different from traditional frame-based neutron cameras where the output is in the form of a matrix of pixel activation levels that is updated at predetermined regular intervals. Instead of information on individual neutron interactions as in the event mode, each of these pixel activation levels (approximately) indicates the local intensity of the incoming neutron flux integrated over a time period within one of the intervals.

While the detector developments are currently focused on neutrons, the principle is also applicable to other particles such as high energy photons (gamma rays and X-rays). In this paper, the new detector concept will be discussed as a general concept for particle detection.

We propose the term “LumaCam” to describe this new type of detector. In the past, LumaCam detectors for neutrons have sometimes been referred to as Timepix detectors after the Timepix3 chip^4^ used for building a large part of these detectors. While renaming the detector type to LumaCam goes against the preferred approach of keeping to established terminology, the term “Timepix detector” is unsatisfying in multiple ways. Other types of neutron detectors are also using a Timepix chip^5–8^, and this has already lead to confusion as to which detector type is being referred to on several occasions in the past. When including detectors for other particles as well, there are many more additional systems using Timepix chips^9–16^. At the same time, the LumaCam detector concept is not limited to using a Timepix chip. LumaCam detectors using other imaging sensors have already been demonstrated on a proof-of-principle level^17,18^.

Within this work, we provide a detailed description of which detectors the term “LumaCam” should encompass and what the general properties of these detectors are (“Detector description” section). To further illustrate the concept and show an example of a working LumaCam detector, we also provide a detailed technical description of an existing neutron-sensitive LumaCam detector (“Example implementation” section), including the complete mathematical description of the event reconstruction used in the readout software (“Event reconstruction” section). To showcase some of the advantages over traditional imaging detectors, we also show some results measured with this detector (“Demonstration measurement” section). An analysis of the performance or possible applications of LumaCam detectors is not part of this document. These topics have to some degree been explored in the past^1,18^ and will also be discussed in future publications.

## Detector description

### Definition

LumaCam detectors are position sensitive area detectors. They have a sensitive area in the form of a scintillator screen and record information about particles hitting this sensitive area. The information is recorded in a spatially resolved manner, i.e. the output contains information where particles hit the sensitive area. LumaCam detectors operate in event mode. This means that the detector output is a list of recognized particle events containing the time and position of their impact on the detector area.

**Fig. 1:**
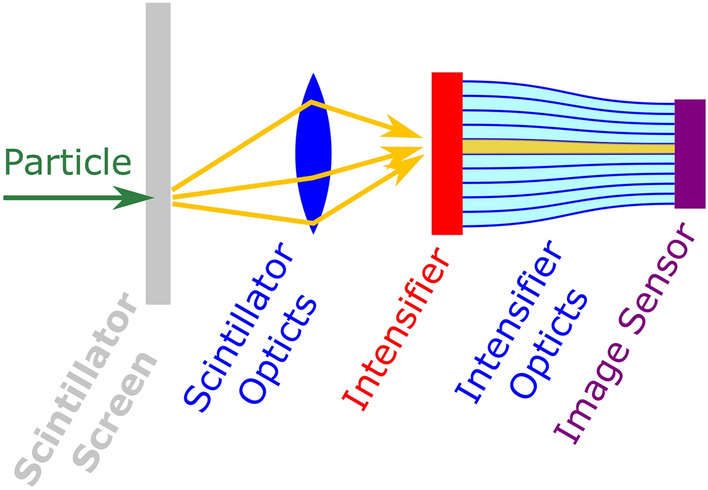
Illustration of the structure of a LumaCam detector. The scintillator and intensifier optics are shown as a lens and a taper only as an example for illustration purposes. Scintillation photons are shown as individual yellow arrows. The intensified light is shown as a solid yellow bar.

The primary components are a scintillator screen, an image intensifier, and an image sensor with the associated readout. Two sets of optics are required. One of them is located between the scintillator screen and the image intensifier (scintillator optics), and the other one between the image intensifier and the image sensor (intensifier optics). There are different components that can be used to realize these optics such as lenses or fiber optic tapers. A schematic overview of the components of a LumaCam detector is shown in Fig. 1.

Incoming particles interact with the scintillator screen and produce scintillation photons. These photons are mapped onto the intensifier by the scintillator optics. The intensifier produces a burst of light at its output for every scintillation photon it captures. This burst is mapped onto the image sensor by the intensifier optics. The amount of light in a burst from a single scintillation photon is enough to activate the pixels on the image sensor. The entire system is therefore sensitive to individual scintillation photons. A reconstruction algorithm generates the list of particle interaction events for the detector output based on the pixel activation information from the image sensor.

### Comparison to other scintillator-screen based imaging detectors

The structure of a LumaCam detector is similar to what can be found in traditional scintillator-screen based neutron imaging detectors^2^. However, there are some key differences. In terms of components, the core feature that separates a LumaCam detector from a traditional scintillator-based imaging detector is that the image sensor in a LumaCam is fast enough to resolve the pixel activations from different particle events. The exact requirement on the sensor for this criterion depends on the spatial and temporal density of the neutron interactions in the scintillator with higher numbers of neutrons requiring better timing resolution. This allows pixel activation data recorded by the image sensor to be used to reconstruct the original interaction event of the individual particles with the scintillator. The event reconstruction algorithm itself is a feature that is only present in LumaCam detectors, not in traditional scintillator-based neutron imaging detectors. The type of data output is also different. The traditional detectors operate in a frame-based mode where the output is in the form of a matrix of pixel intensity levels (i.e. an image of grey values) that is updated at predetermined regular intervals. This is in contrast to the event-mode list produced by LumaCam detectors.

### Event reconstruction

A single incident particle interaction in the scintillator screen usually produces multiple pixel activations in the image sensor. Several factors contribute to this phenomenon. The scintillator usually emits many photons per incident particle, the emission of which is distributed over a volume inside the scintillator. This distribution can be broadened by effects such as light scattering inside the scintillator screen and slightly out-of-focus optics, and usually covers multiple pixels of the image sensor. In addition, the scintillator decay results in a temporal distribution of the emitted scintillation photons, leading to the possibility of pixels being activated multiple times in succession in response to a single incident particle. Dependent on the exact detector design, only some of these effects may be relevant.

LumaCam detectors use many different kinds of algorithms to reconstruct individual particle interaction events from the pixel activations. However, some generally valid observations can be made. The event reconstruction from multiple activated pixels can frequently be used to produce interaction locations with sub-pixel accuracy. By analyzing the space and time distribution of pixel activations within a single event, noise can be suppressed and, in combination with some scintillators, particle discrimination is possible. An example for this is the neutron/gamma discrimination based on the temporal event shape for ZnS:Ag/$^6$LiF scintillators. It is of course only possible to take advantage of this if the spatial and temporal resolution of the detector components is fine enough to not only separate individual events but also to observe their inner structure. The exact requirements are strongly detector dependent, with the scintillator screen and the optics, but also the desired detector capabilities being highly relevant. The advantages that can be leveraged from the reconstruction are so significant that even detector systems that can in principle be configured to only produce a single pixel activation per particle interaction frequently use e.g. purposely out-of-focus optics to get multiple pixel activations and thereby add a detectable structure to each event.

One technique that is frequently used in reconstruction algorithms for LumaCam detectors is to first reconstruct individual scintillation photons hitting the image intensifier (yellow arrows in Fig. 1) based on the pixel activations in the image sensor. The particle interaction (tip of the green arrow in Fig. 1) is then reconstructed only based on the scintillation photons. This approach essentially follows the physical process in the LumaCam detector backwards. There is a lot of flexibility in the actual implementation of the two parts of the algorithm, and several concepts for these exist (such as the clustering-based approach described in “Event reconstruction” section). One of the advantages of splitting the reconstruction algorithm in this way is that it simplifies the changes that need to be implemented in the reconstruction algorithm if changes are made to parts of the detector. If the scintillator screen or the scintillator optics are changed, only the part of the algorithm that reconstructs the particle interaction based on the scintillation photons needs to be changed. In the same way, changes to the intensifier, intensifier optics, or image sensor only necessitate changes in the reconstruction of the scintillation photons based on the pixel activations. Another advantage is that the intermediate step of the scintillation photons is a physical quantity and can therefore be measured using other devices and even be artificially generated as a more controlled input. This can be used to optimize and test the two parts of the algorithm separately. An example for this would be the use of a short-pulsed laser to generate well-defined photons at the input of the image intensifier to test the capability of the first part of the algorithm to reconstruct these photons from the pixel activations.

## Example implementation

To illustrate the LumaCam concept, a detector was built and brought to the ISIS pulsed neutron source to produce an epithermal neutron time of flight (ToF) image of a silver test sample arrangement. Epithermal neutron ToF was chosen as an application because the absorption resonance patterns in this energy range produce distinctive features in the ToF spectrum that require a relatively good timing accuracy to be well resolved. It is therefore a good application to demonstrate the detector timing accuracy.

### Detector components

**Fig. 2:**
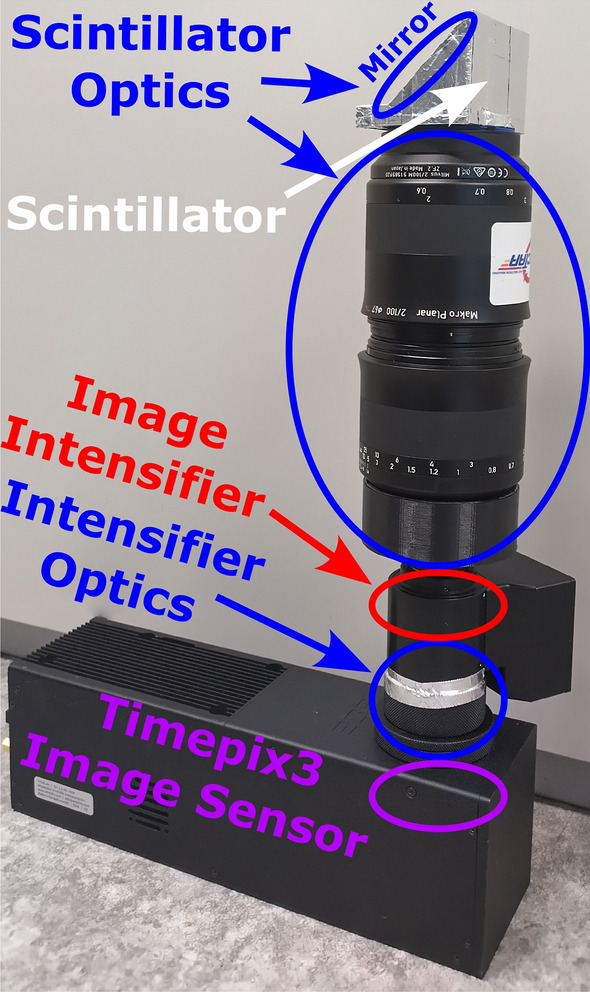
Picture of the LumaCam used for the demonstration measurements. The individual components are highlighted.

Figure 2 shows the LumaCam detector where the individual components are marked. The image sensor used in the detector is made of high resistivity silicon with backside illumination. The depleted sensor has high quantum efficiency in the 400–900 nm wavelength range^19,20^. It is bump-bonded to the Timepix3 chip^4^ mounted on a SPIDR (Speedy PIxel Detector Readout) board^21^ with the entire system provided by Amsterdam Scientific Instruments^22^. The sensor features a 14 mm$\times 14$ mm sensitive area consisting of a grid of $256\times 256$ pixels with a size of 55 µm $\times 55$ µm each. The pixels are sensitive to visible light, requiring ${\gtrsim }\,1000$ photons to activate. The Timepix3 chip is read out in event mode i.e. when pixels are activated, the information on these pixel activations is sent to the output. This includes the position of the pixel on the sensor, the time the activation occurred with a 1.5625 ns precision, and the time over threshold (time for which the pixel was active). The time over threshold is roughly proportional to the number of photons that activated the pixel. No output is generated for pixels that are not activated (sparse readout). This approach is what allows the high timing precision on the pixel activations that is necessary to separate individual events as is required for a LumaCam detector (see “Detector description” section).

The image intensifier is a Photonis Cricket2 from Exosens^23^ with the HI-QE green photocathode, double MCP, and P46 phosphor configuration. The Cricket2 also includes the intensifier optics in the same casing. The intensifier has an analog input used to control the amplification. Applying 0 V to this input results in the highest possible amplification and applying 5 V results in no output. The voltage was set to 0.63 V, resulting in a high enough amplification to activate multiple pixels on the image sensor from a single photon on the intensifier input. The intensifier can be gated down to 3 ns, but this option was not used in the measurement and the intensifier was on during the entire measurement. The input and output areas of the image intensifier are circles with a diameter of 18 mm, and the integrated intensifier optics has a magnification of 1:1. When mapping the circular output of the intensifier to the square image sensor area with these optics, the corners of the image sensor are not illuminated by the image intensifier. At the same time some of the image intensifier area around the edges is mapped to a region outside the sensor area. The useful area is therefore the intersection of the 14 mm $\times 14$ mm square and the 18 mm diameter circle. This configuration is a compromise between maximising the utilization of the areas of the image intensifier and the sensor, necessitated by the incompatible shapes. To simplify the terminology, anything mapped to the 14 mm $\times 14$ mm square is considered to be within the field of view (FoV) for the discussion from here onwards.

The scintillator optics consists of two consecutive lenses and a mirror. The lens mounted close to the intensifier has a focal length of 50 mm and the one mounted close to the scintillator has a focal length of 100 mm. This lens combination results in a 1:2 de-magnification and therefore a 28 mm $\times 28$ mm FoV. The mirror is placed at a $45^\circ$ angle between the lenses and the scintillator.

The scintillator screen consists of a 450 µm thick mixture of zinc oxide doped with additional zinc (ZnO:Zn) and lithium-6 enriched lithium fluoride ($^6$LiF). This scintillator has already been used in wavelength-shifting fiber detectors for neutron diffraction instead of the more conventional zinc sulfide doped with silver (ZnS:Ag) to reduce problems associated with afterglow^24^. The screen covers an area of 30 mm $\times 30$ mm, completely filling the FoV with a 1 mm margin. Due to the $45^\circ$ angle of the mirror, the normal vector of the scintillator screen is at a $90^\circ$ angle to the optical axis of the scintillator optics, allowing all major detector components apart from the scintillator and the mirror to be placed outside the direct neutron beam hitting the scintillator. This is a configuration commonly found in traditional scintillator-based imaging detectors as well to reduce noise from scattering and direct sensor hits, as well to limit activation and radiation damage for the detector components.

### Event reconstruction

**Fig. 3:**
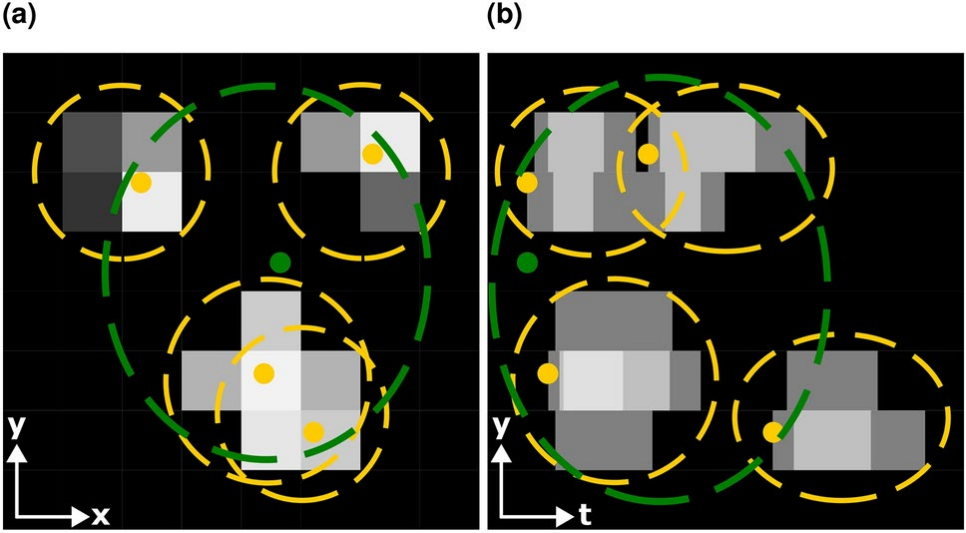
Illustration of the event reconstruction algorithm. (**a**) Shows the pixel activations integrated in time with lighter grey values indicating pixels that are active for longer. Completely inactive pixels are shown in black. (**b**) Shows the pixel activations integrated in x direction with lighter grey values indicating more pixels being active at the same time. The first algorithm step is shown in yellow with the identification of the pixel activations belonging to separate scintillation photons as dashed dashed lines and the reconstructed scintillation photons as dots. The second reconstruction step is shown in green with the identification of scintillation photons belonging to the same neutron event as a dashed line and the reconstructed neutron interaction as a dot.

As explained in “Event reconstruction” section, the event reconstruction algorithm analyzes the pixel activations registered by the Timepix3 sensor and reconstructs the events (in this case neutron interactions in the scintillator) from this data. It is based on the two stage approach also described in “Event reconstruction” section. The first stage of the algorithm identifies the pixel activations belonging to separate scintillation photons and reconstructs the scintillation photons from them. The second reconstruction step identifies scintillation photons belonging to the same neutron event and reconstructs the neutron interaction from them. This is illustrated in Fig. 3. The following notation will be used throughout the rest of this publication to describe the different steps of the algorithm in detail:

- $x_a$: The x-coordinate of a pixel activation, photon, or event *a*.
- $y_a$: The y-coordinate of a pixel activation, photon, or event *a*.
- $t_a$: The arrival time of a pixel activation, photon, or event *a*.
- $\tau _a$: The time over threshold of a pixel activation *a*.

#### Scintillation photon reconstruction

The first step of the algorithm is to group the pixel activations. The grouping is done using the hierarchical single linkage clustering method^25^. Hierarchical single linkage clustering always merges the two groups which are closest together, starting with one group for every element in the original set, containing only the corresponding element. The distance between groups is defined as the smallest distance between two elements of the two groups. How the distance between elements is calculated is problem-specific and defined by a distance function. The merging continues until all elements are in the same group, increasing the distance between the groups that are being merged with every step. By keeping track of the distance between the two groups in a merge (called height), a so-called tree can be constructed, containing every merge with the height it occurred at. By cutting the tree at a specific height, all merges higher up than this height are removed and the groups that are left are the result of the clustering.

Here, the distance function $d_\text {px}\left( p,p'\right)$ for two pixel activations *p* and $p'$ depends on two parameters: the maximum spatial distance $d_{\text {px},s}$ and the maximum temporal distance $d_{\text {px},t}$. It is defined as

1 $$\begin{aligned} d_\text {px}\left( p,p'\right) = \text {max}\left( \frac{\sqrt{\left( x_{p'} - x_p \right) ^2 + \left( y_{p'} - y_p \right) ^2}}{d_{\text {px},s}}, \frac{\vert t_{p'} - t_{p} \vert }{d_{\text {px},t}} \right) , \end{aligned}$$

and the resulting tree is cut at a height of 1. This means that pixel activations that are at most $d_{\text {px},s}$ apart in space and at most $d_{\text {px},t}$ apart in time are put in the same cluster. The grouping of the pixel activations is illustrated with dashed yellow lines in Fig. 3. It should be noted that the two groups on the bottom are not separated in the time integrated view in Fig. 3a. They can only be recognized as separate groups due to ability of resolving their separation in time, as shown in Fig. 3b.

Let *H* be the set of all clusters, i.e. each element *A* in *H* is a set containing exactly the pixel activations in one of the clusters. Clusters with a lower number of pixel activations generally have a higher chance of being caused by image sensor noise and provide poorer results for the subsequent reconstruction of the photon position. For this reason, the clusters are filtered, accepting only clusters containing at least *k* pixel activations:

2 $$\begin{aligned} H' = \{A \in H \mid \vert A \vert \ge k \}. \end{aligned}$$

For each cluster *A* in $H'$, a corresponding scintillation photon $a\left( A\right)$ is reconstructed with the following properties (illustrated as yellow dots in Fig. 3):

3 $$\begin{aligned} \begin{aligned} x_{a\left( A\right) }&= \frac{\sum \limits _{p \in A}x_p \cdot \tau _p}{\sum \limits _{p \in A}\tau _p}, \\ y_{a\left( A\right) }&= \frac{\sum \limits _{p \in A}y_p \cdot \tau _p}{\sum \limits _{p \in A}\tau _p}, \\ t_{a\left( A\right) }&= \text {min}\left( \{t_p \mid p \in A \} \right) . \end{aligned} \end{aligned}$$

As the time over threshold is roughly proportional to the number of photons that activated the pixel, the calculated *x* and *y* coordinates are an approximation of the average position of the intensified light on the image sensor. It should be noted that this average position is not fixed to the pixel grid but is (almost) a continuous value due to the large number of possible combinations of pixel activation arrangements and time over threshold values. In general, not all pixel activations in a single group are registered at the same time. As it is impossible for a scintillation photon to produce a pixel activation before it arrives, the earliest pixel activation is closest in time to the real arrival time of the scintillation photon, and its time is therefore used as the reconstructed scintillation photon time.

#### Event reconstruction from scintillation photons

The steps to reconstruct the neutron interaction events from the scintillation photons are similar to the steps used to reconstruct the scintillation photons from pixel activations. The first step is grouping the pixel activations using the same hierarchical single linkage clustering method. The clustering distance function $d_\text {ph}\left( p, p' \right)$ for two photons *p* and $p'$ depends on two parameters: the maximum spatial distance $d_{\text {ph},s}$ and the maximum temporal distance $d_{\text {ph},t}$. It is defined as

4 $$\begin{aligned} d_\text {ph}\left( p,p'\right) = \text {max}\left( \frac{\sqrt{\left( x_{p'} - x_p \right) ^2 + \left( y_{p'} - y_p \right) ^2}}{d_{\text {ph},s}}, \frac{\vert t_{p'} - t_{p} \vert }{d_{\text {ph},t}} \right) , \end{aligned}$$

and the resulting tree is cut at a height of 1. This means that photons that are at most $d_{\text {ph},s}$ apart in space and at most $d_{\text {ph},t}$ apart in time are put in the same cluster. A single cluster of photons is illustrated in Fig. 3 as a green dotted line.

Let $G^*$ be the set of all clusters, i.e. each element *A* in $G^*$ is a set containing exactly the scintillation photons in one of the clusters. If a cluster $A \in G^*$ spans a time larger than a predefined maximum event duration $D_t$ (i.e if $\text {max}\left( \{ t_a \mid a \in A \}\right) - \text {min}\left( \{ t_a \mid a \in A \}\right) \le D_t$), it is split in two clusters $A'$ and $A''$ with $A'$ containing all photons within the first $D_t$ time span and $A''$ containing the remaining photons:

5 $$\begin{aligned} \begin{aligned} A'&= \{ a' \in A \mid a' - \text {min}\left( \{ t_a \mid a \in A \}\right) \le D_t \}, \\ A''&= A \setminus A', \\ G^*&\leftarrow \left( G^* \setminus \{A\} \right) \cup \{ A', A'' \}. \end{aligned} \end{aligned}$$

This is repeated until there are no clusters spanning a time larger than $D_t$ left in $G^*$. This set is then called *G*. This splitting of clusters limits the maximum extent a single cluster can have and thereby reduces the probability of a single cluster containing multiple particle interaction events which would only be counted as a single event if combined in one cluster.

Clusters containing lower numbers of reconstructed photons have a higher chance of being the result of detector noise, scintillator afterglow or gamma interactions, instead of belonging to real neutron interaction events. In addition, clusters containing lower numbers of reconstructed photons also result in a worse result of the subsequent time and position reconstruction steps. The clusters in *G* are therefore filtered, accepting only clusters containing at least *m* scintillation photons:

6 $$\begin{aligned} G' = \{A \in G \mid \vert A \vert \ge m \}. \end{aligned}$$

For each cluster *A* in $G'$, a corresponding neutron event $a\left( A\right)$ is reconstructed with the following properties (illustrated in Fig. 3 as a green dot):

7 $$\begin{aligned} \begin{aligned} x_{a\left( A\right) }&= \frac{\sum \limits _{p \in A}x_p}{\vert A \vert }, \\ y_{a\left( A\right) }&= \frac{\sum \limits _{p \in A}y_p}{\vert A \vert }, \\ t_{a\left( A\right) }&= \text {min}\left( \{t_p \mid p \in A \} \right) . \end{aligned} \end{aligned}$$

This results in the set of all reconstructed events, which is the output of the event reconstruction algorithm:

8 $$\begin{aligned} G'' = \{ a\left( A\right) \mid A \in G' \}. \end{aligned}$$

### Demonstration measurement

The epithermal neutron ToF measurements were performed at the EMMA beamline^26^ at the first target station (TS1) of the ISIS neutron and muon source^27^ in the UK. At TS1, neutrons are produced from a proton-induced spallation reaction in a tantalum-clad tungsten target. The protons are delivered by an accelerator in short pulses with a frequency of 50 Hz (however, every 5th pulse is missing as it is redirected to the second target station). Each accelerator proton pulse produces a neutron pulse, which is moderated in a 300 K thermal water moderator before arriving at the EMMA beamline. The T0 chopper of EMMA that is normally used during thermal neutron measurements was turned off during this measurement to increase the epithermal neutron flux.

**Fig. 4:**
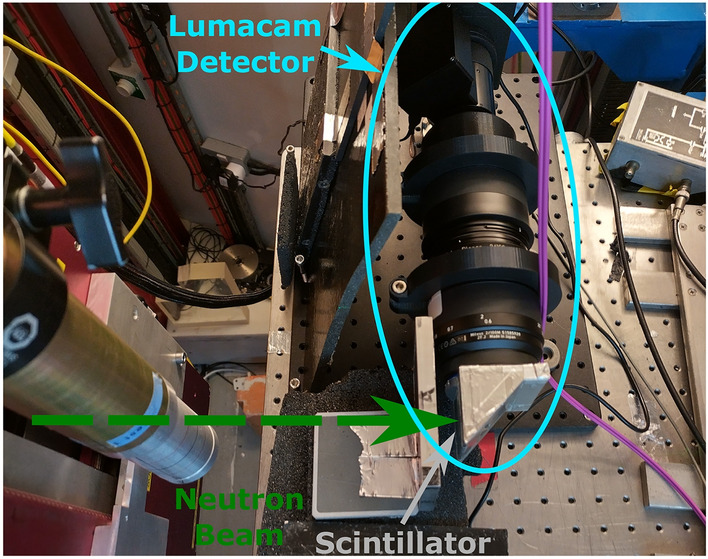
Picture of the LumaCam detector set up at the EMMA beamline for the open beam measurement. The sample was attached to the detector directly in front of the scintillator for the measurement with the sample.

**Fig. 5:**
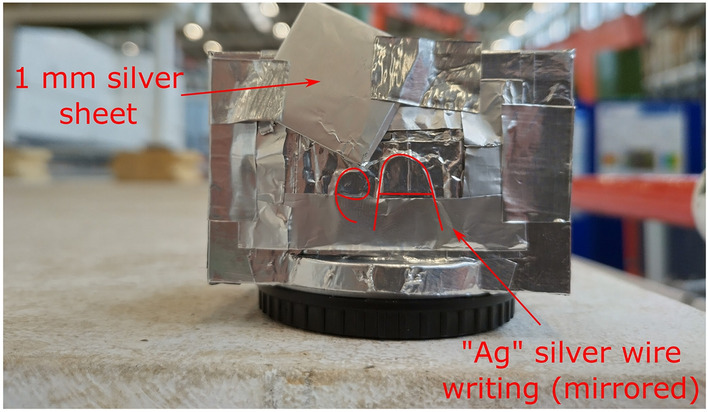
Arrangement of the test sample attached to the head of the detector. The writing is mirrored to appear in the correct orientation when viewed by the detector.

Figure 4 shows the LumaCam detector installed on the beamline. The distance from the source to the detector was ${\sim }16.5$ m. Two measurements were acquired. The first one was an open beam measurement without a sample. The second one had two samples, a silver sheet and a set of silver wires forming the letters ”Ag”. Both samples were directly attached to the detector in front of the scintillator. Figure 5 shows the detached head of the detector (containing the mirror and the scintillator) with the samples attached. Timestamps of the neutron pulses from the ISIS source were recorded during both measurements to calculate the ToF and perform normalization.

**Table 1:** Values used for the different parameters during event reconstruction.

Variable	Symbol	Value
Maximum pixel activation distance space	$d_{\text {px},s}$	1 px
Maximum pixel activation distance time	$d_{\text {px},t}$	50 ns
Minimum pixel activation number	*k*	2
Maximum photon distance space	$d_{\text {ph},s}$	3 px
Maximum photon distance time	$d_{\text {ph},t}$	1 µs
Maximum event duration	$D_t$	5 µs
Minimum photon number	*m*	3

The algorithm described in “Event reconstruction” section was used to reconstruct the neutron events. The parameter values are listed in Table 1. For each of the resulting events the ToF was calculated by subtracting the time of the most recent neutron source pulse from the time of the event. The events were binned in position and ToF, creating a stack of 512 images, each corresponding to a 1.5625 µs ToF interval. For further analysis, the first 80 images were removed, narrowing the ToF window to between 130 and 800 µs, which corresponds to an energy interval of ${\sim \,}2$  eV to ${\sim\, } 80$  eV. In this energy region, the neutron transmission spectrum of silver is dominated by so-called absorption resonances. These are narrow energy regions for which the transmission drops due to an increased absorption cross-section for neutrons at that energy. As silver is close to transparent to neutrons for energies between these resonances, they provide an excellent contrast in a ToF spectrum.

The transmission value $T_{px, t}$ for each pixel *px* in each slice *t* is calculated using the following equation:

9 $$\begin{aligned} T_{px,t} = \frac{\frac{1}{P_S} \cdot S_{px,t}}{\frac{1}{P_O} \cdot O_{px,t}}. \end{aligned}$$

**Fig. 6:**
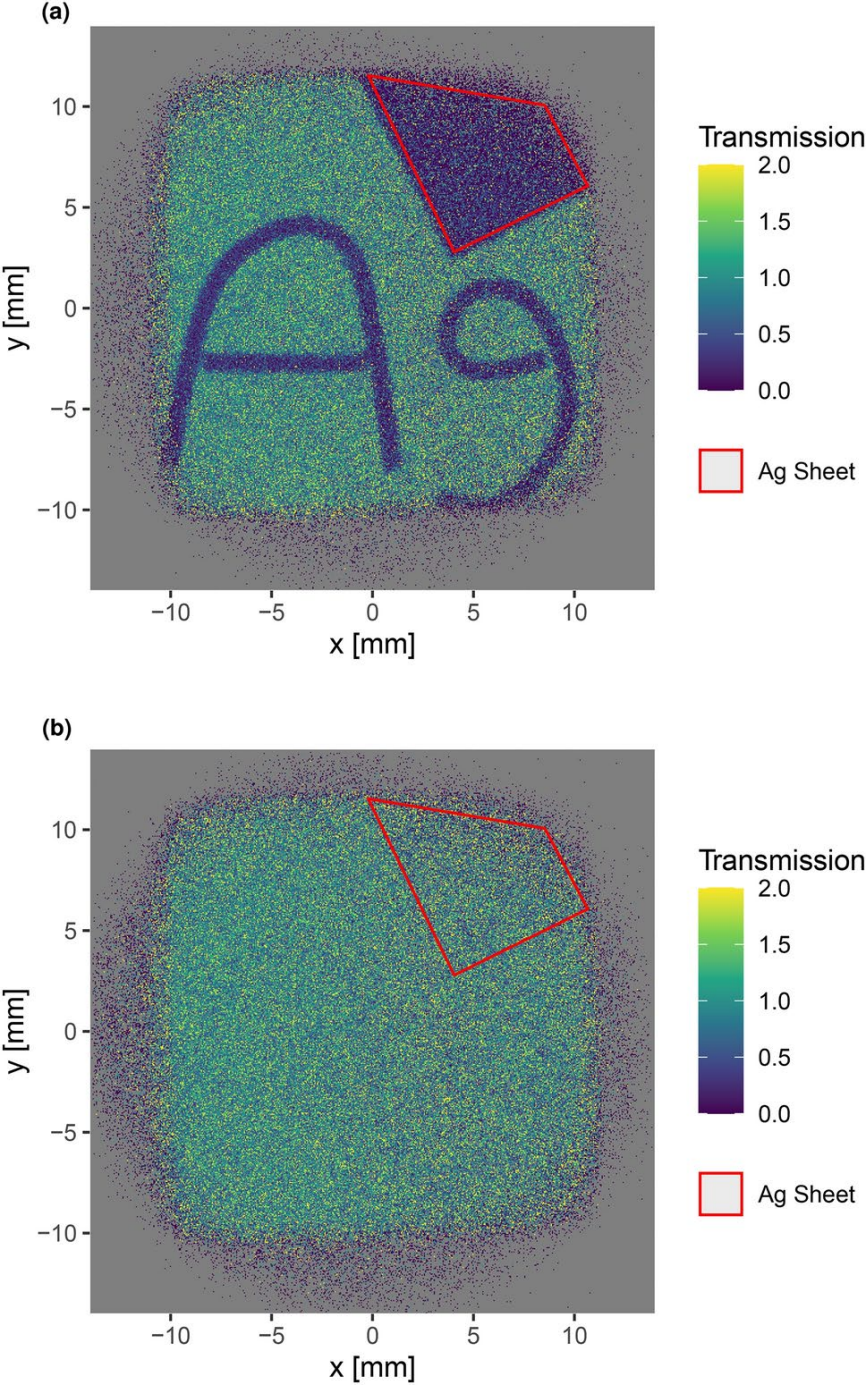
Radiograph of the test sample in the lowest energy resonance interval (**a**) and in an off-resonance region (**b**). The samples are clearly visible in (**a**), including small details, but almost completely invisible in (**b**). The silver sheet region used to produce the data displayed in Fig. 7 is shown in red. The grey region along the edges of the images is outside the neutron beam.

**Fig. 7:**
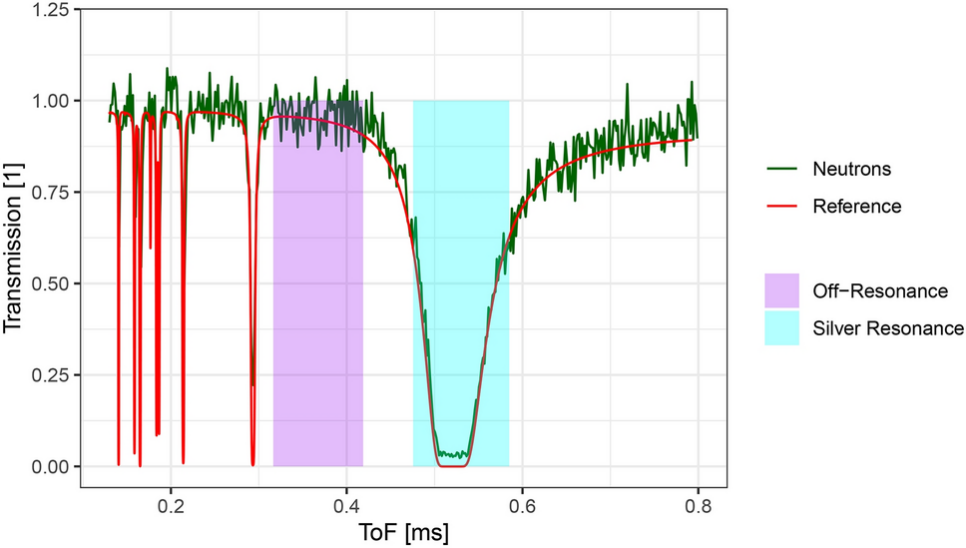
Transmission spectrum measured in the region of the silver sheet (green) and calculated from literature data (red). The two lines agree well with the measured data having a 3% transmission in the center of the lowest energy resonance. The two intervals used to generate Fig. 6 are shown in violet and cyan.

Here, $S_{px,t}$ denotes the number of events in a pixel *px* and slice *t* measured with the samples in pace, and $O_{px,t}$ denotes the number of events in the same pixel and slice measured without the samples. $P_S$ and $P_O$ are the number of source pulses during the measurement with and without the samples respectively. No background subtraction is performed. Figure 6 shows the resulting transmission image for the ToF interval in the lowest energy (highest ToF) silver resonance region (Fig. 6b), and for the ToF interval directly before the resonance (Fig. 6a). The corresponding ToF intervals are shown in Fig. 7.

In the resonance region, the sample is clearly visible with a high contrast. This includes small features such as gaps between the wires used to form the “Ag” writing. In contrast to this, the sample is almost completely invisible in the off-resonance region. This shows that the two regions are well separated in the detector output.

Figure 7 shows the transmission spectrum measured in the silver sheet region of the image. As a comparison, the theoretical transmission spectrum for 1 mm of silver with a natural isotopic composition is shown. The theoretical transmission is calculated from the total neutron interaction cross section (N,TOT) data from the ENDF/B-VIII.0^28^ library for $^{107}$Ag and $^{109}$Ag. The transmission is calculated using the Beer-Lambert law, i.e. without considering the possibility that scattered neutrons can still hit the detector. The theoretical transmission spectrum is matched well by the measured data.

The lowest energy resonance has a region where the theoretical transmission is essentially zero. The measured transmission spectrum in this region is approximately flat at 3%. Since the measured transmission is flat, the detector counts in this region cannot be due to (small) timing inaccuracies. These counts can therefore be used as an estimate of the constant noise of the setup. However the noise contribution of the detector itself (i.e. from dark noise and gamma rays) is lower than this estimate as scattered neutrons are contributing to it as well.

**Fig. 8:**
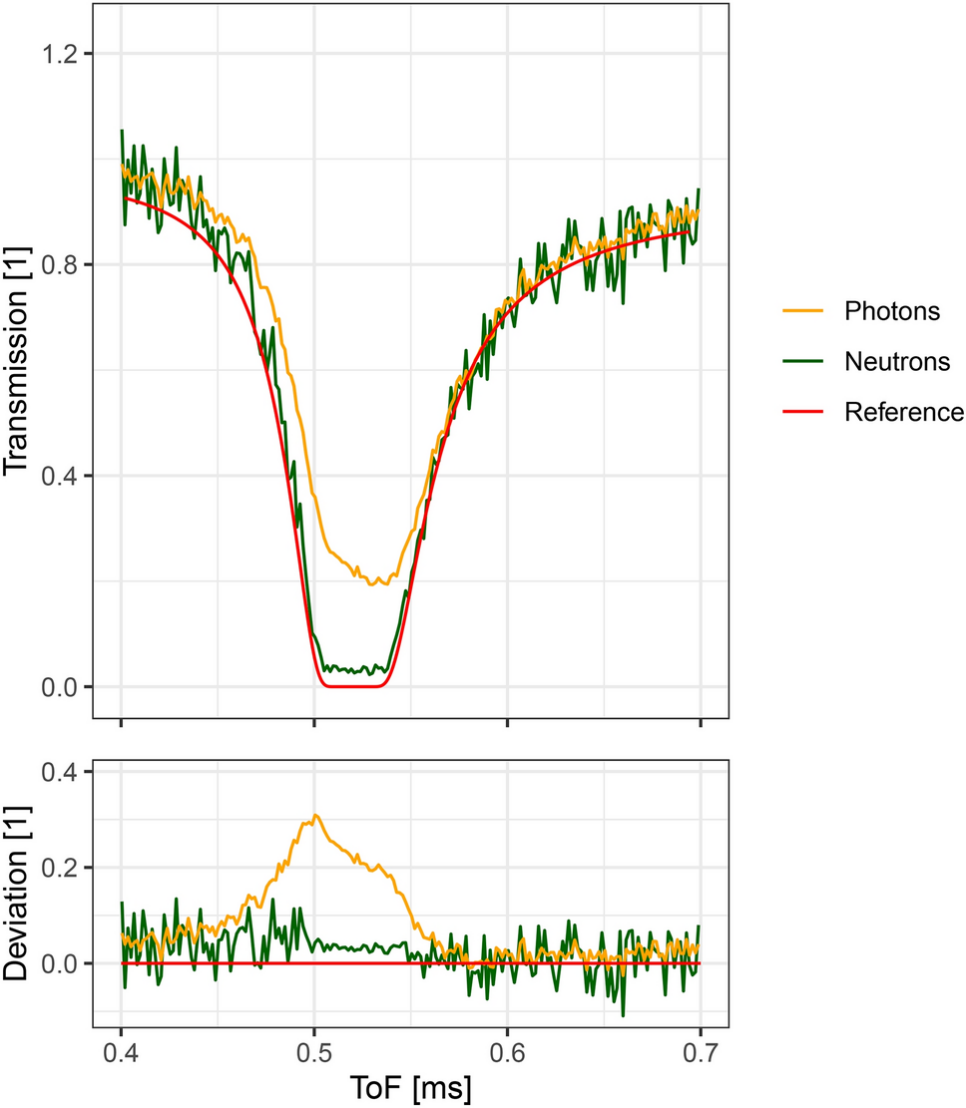
Comparison of the transmission spectrum for the lowest energy silver resonance calculated from measured neutrons and photons in the region of the silver sheet. While the transmission calculated from the neutron event data closely follows the reference data, the transmission calculated from the photon event data displays a large deviation. It lags behind and exhibits a decay behavior in the region where the reference is flat close to 0. The photon transmission only reaches down to 20% at the lowest point, several times higher than the 3% of the neutron transmission.

Figure 8 shows the transmission calculated from the neutron event data compared to the transmission calculated from the scintillation photon event data, i.e. the output of just the first step of the event reconstruction algorithm without applying the second step. While the transmission from the neutron event data follows the reference data at the falling and rising edge of the resonance, the transmission calculated from the photon event data has a significant delay for the falling edge. This continues in the center of the resonance where, instead of being flat and close to zero, the transmission calculated from the photon data shows a strong decay behavior and only reaches 20% transmission at the lowest point. This behavior is due to the decay of the scintillator and demonstrates the capabilities of the second step of the reconstruction algorithm to improve the temporal resolution of the detector by almost completely eliminating the scintillator decay effect. It also demonstrates the inherent advantage of event mode neutron detection over classical neuron imaging when using scintillators. As classical imaging detectors simply integrate the light from the scintillator, they are inherently limited in their timing resolution by the scintillator decay, even if the frame rate of the image sensor would allow a better timing accuracy.

## Conclusion and outlook

We have provided a detailed technical and mathematical description of a new type of position sensitive particle detectors that operate in event mode. Although these detectors have originally been developed for neutron applications, they can also be used to detect other particles such as high energy photons (X-rays and gammas). We suggest the term LumaCam for the detectors to facilitate communication and avoid confusion with other detector types. To further illustrate the LumaCam concept, an example detector was built and used to collect a high resolution energy resolved epithermal neutron radiograph via ToF imaging. The resulting images are very clean and feature a good time resolution. The absolute transmission values closely follow the theoretical transmission calculated from literature values. The transmission profiles also clearly show the fundamental advantage of LumaCam detectors over traditional scintillator-based neutron imaging detectors with respect to temporal accuracy.
